# Development of an Online Well-Being Intervention for Young People: An Evaluation Protocol

**DOI:** 10.2196/resprot.4098

**Published:** 2015-04-30

**Authors:** Gaston Antezana, Niranjan Bidargaddi, Victoria Blake, Geoffrey Schrader, Billingsley Kaambwa, Stephen Quinn, Simone Orlowski, Megan Winsall, Malcolm Battersby

**Affiliations:** ^1^Flinders Human Behaviour & Health Research Unit (FHBHRU)Department of Psychiatry, School of MedicineFlinders UniversityAdelaideAustralia; ^2^Young and Well CRCMelbourneAustralia; ^3^ReachOut.comSydneyAustralia; ^4^Flinders Health Economics GroupSchool of MedicineFlinders UniversityAdelaideAustralia; ^5^Flinders UniversitySchool of MedicineFlinders UniversityAdelaideAustralia

**Keywords:** well-being, mental health, young people, online intervention, apps, engagement

## Abstract

**Background:**

Research has shown that improving well-being using positive mental health interventions can be useful for predicting and preventing mental illness. Implementing online interventions may be an effective way to reach young people, given their familiarity with technology.

**Objective:**

This study will assess the effectiveness of a website called the “Online Wellbeing Centre (OWC),” designed for the support and improvement of mental health and well-being in young Australians aged between 16 and 25 years. As the active component of the study, the OWC will introduce a self-guided app recommendation service called “The Toolbox: The best apps for your brain and body” developed by ReachOut.com. The Toolbox is a responsive website that serves as a personalized, ongoing recommendation service for technology-based tools and apps to improve well-being. It allows users to personalize their experience according to their individual needs.

**Methods:**

This study will be a two-arm, randomized controlled trial following a wait-list control design. The primary outcome will be changes in psychological well-being measured by the Mental Health Continuum Short Form. The secondary outcomes will be drawn from a subsample of participants and will include depression scores measured by the Center for Epidemiologic Studies Depression Scale, and quality of life measured by the Assessment of Quality of Life-four dimensions (AQOL-4D) index. Cost-effectiveness analysis will be conducted based on a primary outcome of cost per unique visit to the OWC. Utility-based outcomes will also be incorporated into the analysis allowing a secondary outcome to be cost per quality-adjusted life year gained (based on the AQOL-4D values). Resource use associated with both the intervention and control groups will be collected using a customized questionnaire. Online- and community-based recruitment strategies will be implemented, and the effectiveness of each approach will be analyzed. Participants will be recruited from the general Australian population and randomized online. The trial will last for 4 weeks.

**Results:**

Small but clinically significant increases in well-being symptoms are expected to be detected in the intervention group compared with the control group.

**Conclusions:**

If this intervention proves to be effective, it will have an impact on the future design and implementation of online-based well-being interventions as a valid and cost-effective way to support mental health clinical treatment. Findings regarding recruitment effectiveness will also contribute to developing better ways to engage this population in research.

**ClinicalTrial:**

This study is registered in the Australian New Zealand Clinical Trials Registry (ANZCTR): ACTRN12614000710628.

## Introduction

### Background

Positive mental health implies more than just the absence of illness, it relates to *positive symptoms* and emphasizes psychological, social, and generative dimensions such as positive affect, personal growth, social actualization, and social contribution among others [1,2]. Despite controversies surrounding the role of well-being in mental health, the concept of positive mental health is steadily gaining credibility [1]. There is evidence that people with high levels of positive mental health are physically healthier, live longer, are more productive at work, and use less health care [3].

Consistent with this reasoning, the World Health Organization defines mental health as “a state of well-being in which the individual realizes his or her own abilities, can cope with the normal stresses of life, can work productively and fruitfully, and is able to make a contribution to his or her community” [4]. Promoting positive mental health can be an effective strategy to approach mental health comprehensively as it is recognized that “the alleviation of symptoms of illness does not guarantee the presence of wellness” [5]. The Complete State Model of Mental Health characterizes mental health into three stages, namely, flourishing, languishing, or moderately mentally healthy, based on symptoms of positive feeling and functioning in life [6]. Keyes and Lopez [6] have posited that positive mental health constitutes a different continuum to mental illness and although correlated, the absence of mental illness does not imply the presence of positive symptoms [1]. Keyes [3] found that people with diagnoses less than flourishing struggled as much as people with a mental illness in areas such as work, health limitations, and psychosocial functioning [1]. In addition, the absence of positive mental health is associated with an increase in risky behaviors and the potential onset of mental illness [7]. A study conducted in South Australia reported that less than half of South Australian young people (42%) aged 13-17 years had a *flourishing* diagnosis of mental health, with even lower rates reported in rural areas [7]. This means that at the time of the study, 58% of young people in South Australia had less than a complete state of mental health (flourishing), and might therefore be more vulnerable to struggle in one or more areas of their lives.

### Online Well-Being Interventions

There is evidence that young people make significant use of the Internet to socialize and to find information about a wide range of issues including personal difficulties. In Australia, surveys show that the Internet is the preferred medium of support for young people after family and friends [8,9]. Specialized online mental health services for young people such as ReachOut.com have effective reach into this population group [10].

Online treatments based on a psychopathology model for a range of mental disorders such as depression and anxiety have been researched widely [11-14]. Most of these interventions are based on cognitive behavioral therapy (CBT) with specific variations such as prolonged exposure or guided relaxation, among others. These interventions have shown effectiveness in treating problems such as stress, anxiety, and depression. Other types of interventions such as general counseling and self-help approaches still show effectiveness, although to a lesser extent [11,12]

Regarding online positive psychological interventions, a number of studies in adult populations have shown positive results in improving well-being and decreasing depression. Results vary depending on the actual intervention and method of delivery [15]. Two recent trials with similar characteristics to this study demonstrated significant improvements in well-being in the general adult population, predominantly in women. Bolier et al [16] examined the effectiveness of an online self-help intervention consisting of a multicomponent, fully automated self-help online website to improve well-being. Their study investigated mild to moderately depressed adults in the general population using a waitlist control design, and found the online intervention to be effective in enhancing well-being on the 5-item World Health Organization well-being index [17] compared with a control. Small but significant effects were found for general health, vitality, anxiety, and depressive symptoms. Another study comprising a randomized, placebo-controlled, parallel-group trial with longitudinal outcome measurements used the Individual-level Wellbeing Assessment and Scoring Method to measure well-being [18]. It was found that a multimodal online intervention consisting of well-being e-mail, web- and mobile-based interventions had positive effects, with significantly improved well-being in a sample of the general adult population compared with a control.

There is a paucity of research on online positive mental health interventions in young people; however, a randomized controlled trial [19] investigated the effectiveness of a well-being website called “Bite Back” designed for young people aged 12-18 years. Using the Depression, Anxiety and Stress Scale (DASS21)[20] and the Short Warwick Edinburgh Mental Wellbeing Scale [21], the study participants were assigned to two conditions over a 6-week period. The intervention group was given access to the website and the control group was assigned to neutral entertainment-based websites. Results demonstrated significant improvement in clinical measures (depression, anxiety, and stress) and well-being in the participants of the intervention group who reported using the website for at least 30 minutes every week.

### Online Recruitment and Engagement

Facebook, Google, and other online platforms, frequented by large number of users, have been used widely with considerable success to recruit participants for online studies [22-24]. Both Google and Facebook advertising platforms have been effective in recruiting participants with an existing mental health issue for online interventions. The average cost of recruitment is approximately AUD 12-15 for each participant, whereas contacting forums, email groups, and community notice boards are less effective and more costly [24,25]. Although social networking websites can be a promising method to recruit adolescents, the health and behavior patterns of those recruited online could differ significantly from those recruited offline [22]. The evidence for effective strategies to recruit young people for online well-being interventions is sparse. Despite the proven efficacy of Internet-based mental health interventions, they suffer from a high reuse attrition rate [26]. In fact, findings from an online smoking cessation study with a high reach showed that adherence and retention rates decrease as accessibility increases [27]. Improving adherence is a high-priority research area in Internet interventions, as higher usage rates are associated with significant improvements in well-being [19]. Periodic prompts through email, short message service (SMS) text messaging, phone, and online peers/counsellors have proven to be effective strategies in increasing login rates and the time users spend using online interventions [28].

Research has shown that in the first few weeks after engagement with a given intervention, high numbers of participants lose interest [29]. Involving users in the intervention design process can be an effective way to create person-centered innovations [30]. Participatory design is characterized by generative, experiential, and action-based methods that put emphasis on play, co-operative learning, creating visions for the future, and designing-by-doing, with the aim of involving end users in the design of services [31]. Online studies that provide useful feedback and alternative resources for the control group achieve better engagement [18].

### Current Study

This study is a waitlist randomized controlled trial (ACTRN12614000710628) intended to test the effectiveness of an online intervention in improving well-being in a sample of Australian young people aged 16-25 years.

This paper provides a brief description about the Online Wellbeing Centre (OWC), outlining two different configurations for the implementation of interventions and how they were developed. It also includes a description of the methods, design of the trial, and finally a discussion with implications of this study.

## Methods

### Online Wellbeing Centre

**Figure 1:**
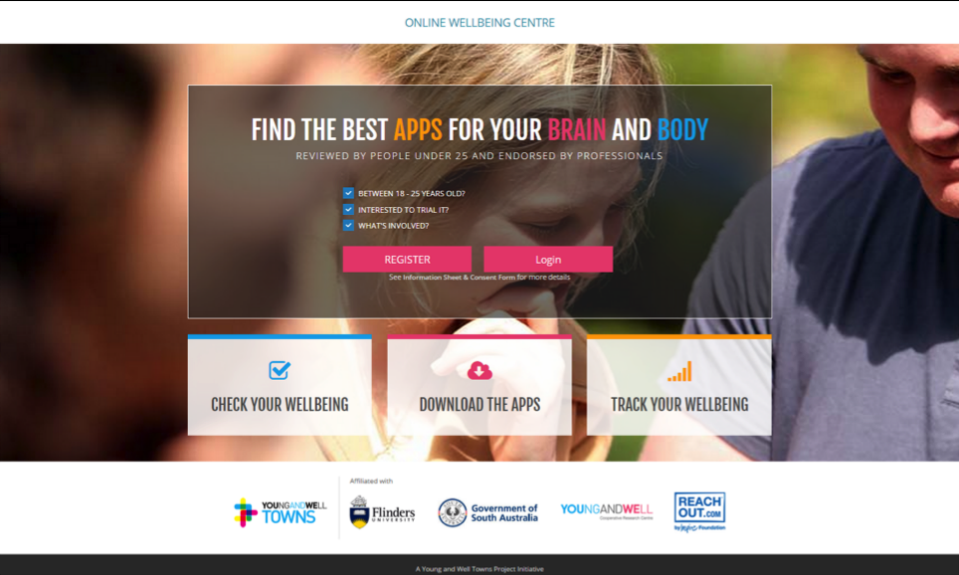
Screenshot of the Online Wellbeing Centre landing page.

**Figure 2:**
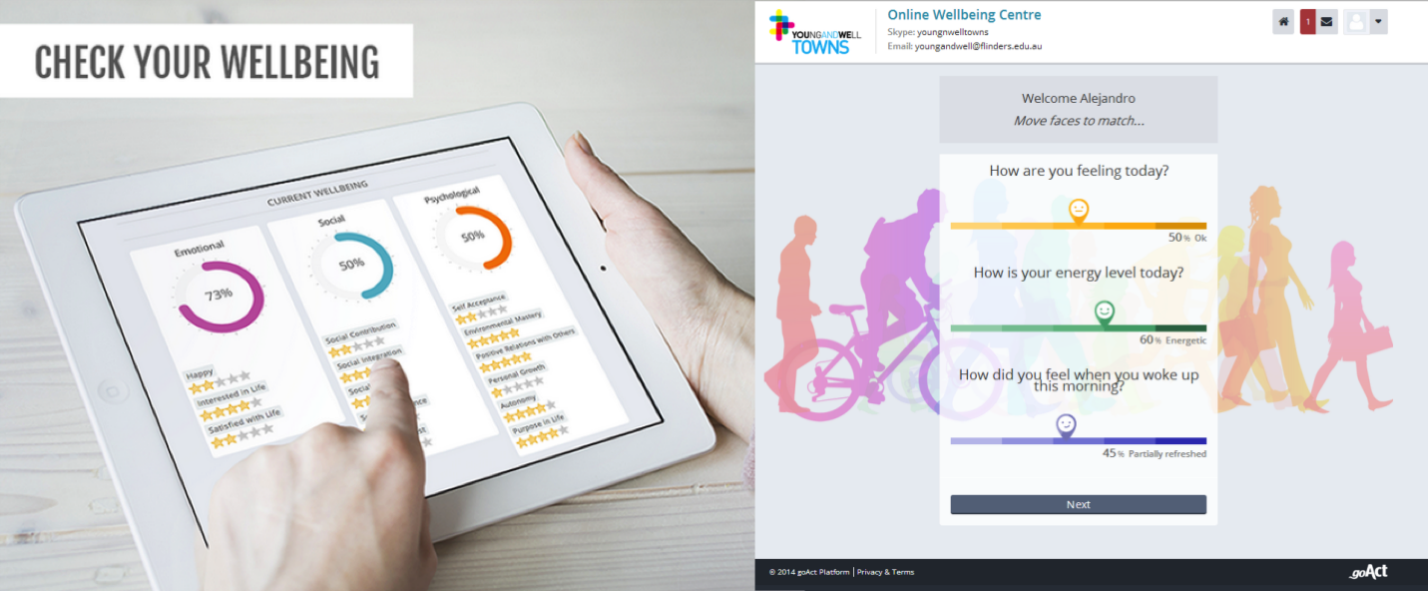
Screenshots of the Online Wellbeing Centre website.

The OWC was designed as a way to encourage individuals to participate in both intervention and control arms. The OWC is a virtual space where users sign up via the landing page (Figure 1), check their well-being, track their progress over time, and access different well-being resources. It has capabilities to randomize, administer study measures at different time points, and give access to tailored resources according to the assigned arms of the study. The OWC provides feedback to users in a meaningful graphic display as shown in Figure 2.

### Intervention arm: “The Toolbox” by ReachOut.com

The Toolbox has been developed by ReachOut.com, with the Young and Well Cooperative Research Centre (Young and Well CRC). It houses over 50 mental health and well-being tools and apps that have been reviewed by a panel of both mental health professionals and consumers for their credibility, functionality, engagement, and aesthetics according to the Mobile Application Rating Scale developed by Queensland University of Technology [32].

**Figure 3:**
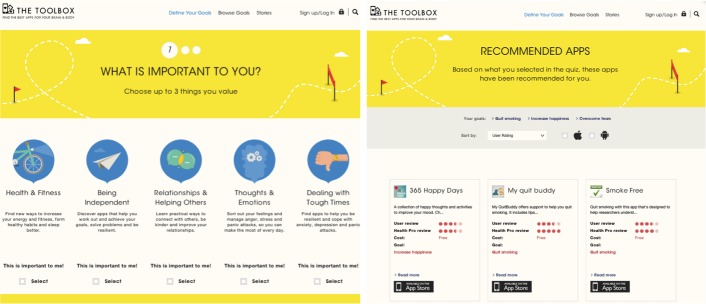
The Toolbox website home page and app recommendation page.

The Toolbox is based on the theory of change proposed by Perugini and Bagozzi’s Model of Goal-Directed Behaviour [33] as well as theories of reasoned and planned behavior [34]. The Toolbox focuses on well-being and positive functioning, and is designed to improve (1) emotional well-being (ie, positive affect and life satisfaction); (2) psychological well-being (ie, self-acceptance, personal growth, purpose in life, environmental mastery, autonomy and positive relations with others); (3) social well-being (ie, social acceptance, social contribution, social coherence, social integration, and social actualization); and (4) physical well-being [35]. In designing The Toolbox, ReachOut.com took a participatory design approach [31]. Young people were consulted at the initiation of the project and during the design phase, while also providing feedback on early prototypes. The user experience of The Toolbox is based on young people’s conceptualization of well-being and what they need from an online recommendation service. Functionalities of The Toolbox website are described in Textbox 1, and screenshots of the first 2 pages of The Toolbox website are shown in Figure 3.

**Functionalities of The Toolbox.**

- Quiz to help participants determine their area of focus and recommend relevant apps.

- Browse apps by category. Categories include Health & Fitness, Being Independent, Relationships & Helping Others, Thoughts & Emotions, and Dealing with Tough Times.

- Read ratings and reviews by health professionals and young people. Participants can write their own review of apps including what they liked and what could be improved.

- Read stories about how others have used apps to achieve their goals and the challenges they faced. Participants can submit their own story.

- Dashboards are available in which participants can update their goals, add new apps, and view their stories.

### Study Design

**Figure 4:**
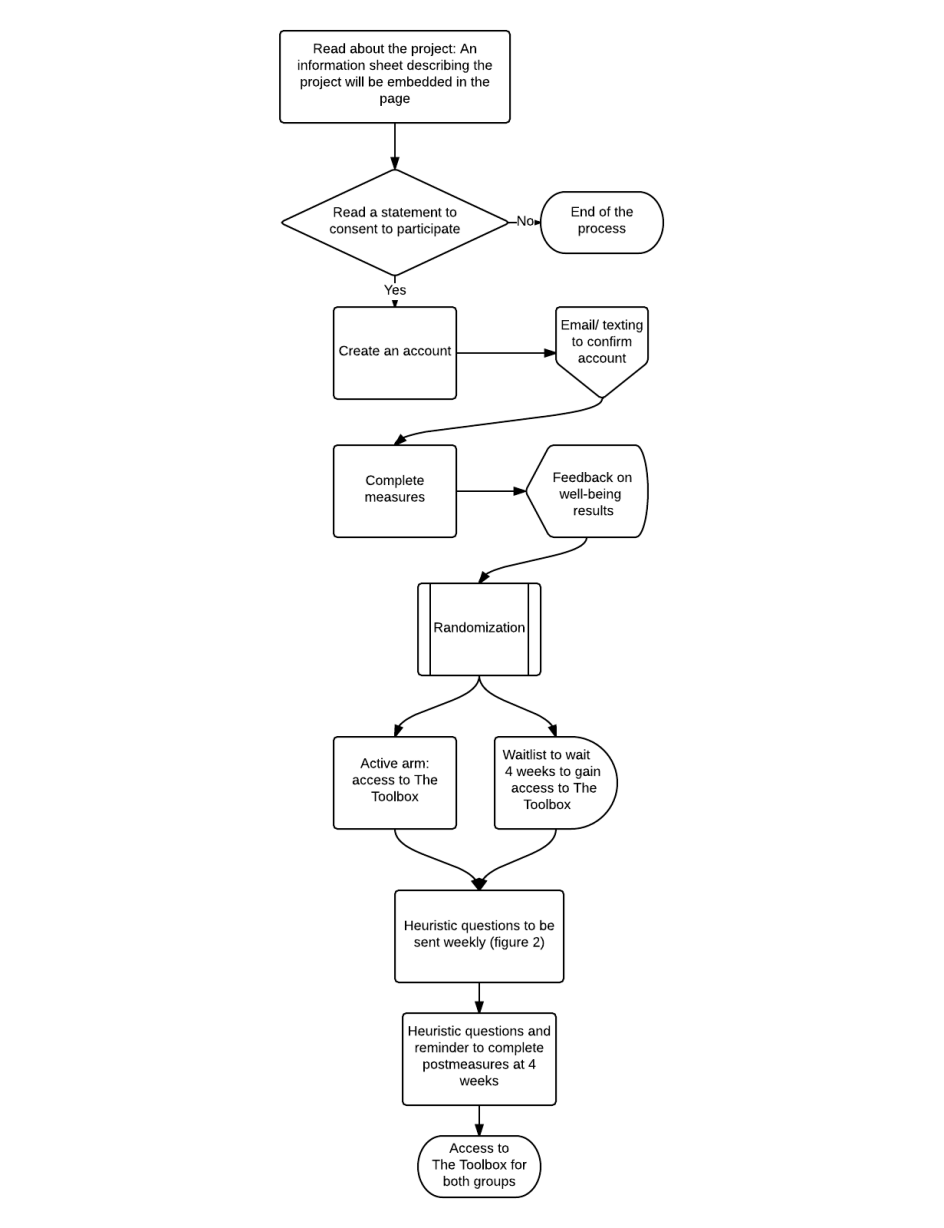
Online Wellbeing Centre study flow diagram.

This is a standard parallel arm clinical trial comparing the effectiveness and cost effectiveness of control and intervention arms offered over 4 weeks. Group 1 represents the intervention arm (OWC with The Toolbox) and Group 2 represents the waitlist control arm (OWC without The Toolbox). In this study, participants in the intervention arm will be able to access the active component of the trial, The Toolbox, through the OWC straight away. Participants in the control group will gain access to The Toolbox after 4 weeks. Once participants follow the online links as described in the recruitment section, they will be directed to the project’s landing page (Figure 1). Once there, they will be required to complete the steps outlined in Figure 4.

All participants will receive regular SMS and email prompts from the OWC to remind them to complete study measures. As detailed in Table 1, assessments will be administered at sign up, and at 4 weeks. In addition, participants will be prompted to regularly monitor their mood, energy and sleep.

**Table 1:** Assessment scales administered at each time point during the study.

Instrument	Measurement	Baseline	After 4 Wk
Mental Health Continuum Short Form	Well-being/Positive mental health	X	X
Center for Epidemiologic Studies Depression Scale	Symptoms of depression	X	X
Assessment of Quality of Life-four dimensions index	Quality of life data	X	X
Resource use questionnaire	Cost effectiveness		X
Demographics	Date of birth, gender, postcode, employment, education, cultural background, and living conditions	X	
Heuristic well-being questions	Feelings, energy, and sleep	Weekly	Weekly

### Recruitment, Inclusion, and Exclusion Criteria

The target population is Australian young people aged between 16 and 25 years, with access to technology and the Internet (computers or smartphones). In the context of well-being theory, pre-existing mental health problems will not be considered as exclusion conditions for this study. Given that this intervention is not intended to treat any particular pathology, all participants could potentially benefit from improving well-being symptoms. Relevant links to appropriate mental health services will be offered to participants during the trial. Online recruitment will only be targeted to participants between 18 and 25 years of age. Parental consent will be obtained for participants under the age of 18 recruited via community channels, given that we will have face-to-face contact with potential participants through schools and other organizations. People not meeting the age criteria and/or residing outside of Australia will be excluded.

**Figure 5:**
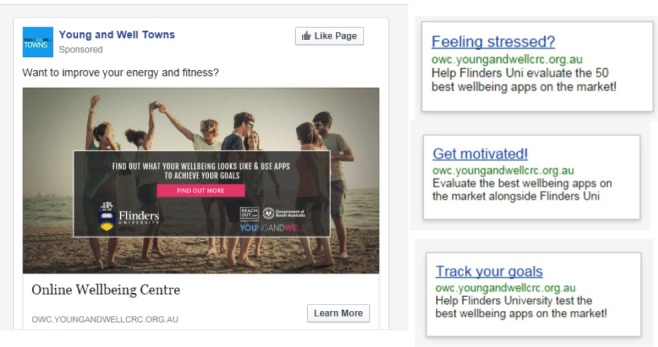
Examples of online Facebook and Google AdWords used in recruitment for study.

Participants will be recruited using online- and community-based methods. Two platforms have been selected from the many options available to implement online paid advertising. Advertisements will be placed on Facebook and Google AdWords (see Figure 5) using keywords relevant to well-being dimensions. The keywords for the advertisements were defined in collaboration with a reference group representing the target population to ensure their validity and relevance. Examples of such keywords include *fitness*, *stress*, *relationships*, *balance*, and *goals*. Concurrently, the links to the study site will be integrated into several websites that are frequently visited by young people from different backgrounds in Australia (most notably the partner organizations of the Young and Well CRC).

The online recruitment strategy also includes changing advertisements periodically. This will be a dynamic process guided and modified by success rates of particular advertisements while they are being implemented. The wording of the advertisements and placement of links will be monitored (according to the method described in the “Recruitment Tracking” section) and periodically modified to reach the target population effectively.

Community-based organizations such as schools, universities, sport clubs, and local councils will be approached and asked to help promote the study. Promotional packages consisting of a video, information, and instructions on how to access the OWC will also be designed. These packages will be distributed to different institutions where they will be presented to potential participants. Innovative technologies (videos, Google Hangouts, etc) will be included in the recruitment packages.

### Recruitment Tracking

To assess the effectiveness of the different recruitment approaches and reach of each advertisement/link, an advertisement tracking system will be implemented using the online open analytics platform *Piwik* [36]. This method involves adding a unique *campaign* and *keyword* code to the source name as a parameter at the end of each link where the advertisements, banners, and links will be embedded.

### Study Hypothesis

The primary hypothesis is that the active intervention group (OWC with The Toolbox) will exhibit an increase in participant well-being [as measured by the Mental Health Continuum Short Form (MHC-SF) scores] after 4 weeks, in comparison with the control group (OWC without The Toolbox).

### Primary Outcome Measure

The primary outcome is mental health (well-being), assessed using the MHC-SF [37]. This scale consists of 14 questions measuring three areas of well-being, namely, emotional, psychological, and social. The tool diagnoses mental health as flourishing, moderately mentally healthy, and languishing, consistent with the Complete State Model of Health theory. The MHC-SF has been validated with adolescents and adults in the United States, South Africa, and the Netherlands. It has an internal consistency over Cronbach alpha = .80 [38-41]. The test-retest reliability of the MHC-SF over three successive 3-month periods averaged .68, and the 9-month test-retest reliability was .65 [40]. A clinically significant change will be defined as a change in diagnostic outcome (languishing, moderately mentally healthy, and flourishing) between measures before and after the intervention in at least one of the well-being categories (emotional, psychological, and social). We will also consider changes in heuristic trackers as reflective of behavioral change (sleep, mood, energy).

### Secondary Outcome Measures

Secondary outcomes include momentary measurements of mood, energy, and sleep using simple visual analog scales ranging from 0 (worst) to 100 (best) at weekly intervals. Changes in these scales will be correlated with the type of apps selected, goals set, and duration spent within the OWC. There is evidence that depression is a significant covariate in analyses of changes in well-being in multiple studies [16,19,42]. Participants who score below 50 for mood in the visual analog scale will be invited to undergo a screening for depression using the Center for Epidemiologic Studies Depression Scale (CESD)[43,44]. Changes in well-being of this subset will be correlated with improvement in depression. To facilitate the cost-effectiveness analysis, data on health-related quality of life will be collected using the Assessment of Quality of Life-four dimensions (AQOL-4D) index [45]. The AQOL-4D includes four dimensions, namely, independent living, mental health, senses, and relationships. Each dimension has three items and ranges from -0.04 to 1.00 with higher score indicating better quality of life. The AQOL-4D is a self-completed questionnaire that can be administered online.

### Subsidiary Outcome Measures

Resource use associated with both the intervention and control groups will be collected from a sample of 50 participants at the end of the trial using a resource use questionnaire. This is a custom questionnaire developed by the funding body Young and Well CRC and includes questions corresponding to the period of the trial on participants’ employment/education status, wages, their use of health services (type and duration), number of times (and duration) they accessed the OWC and other mental-health-related Internet sites, and how much they pay to access the Internet.

### Sample Size

The primary outcome measure is change in well-being as measured by MHC-SF. A total of 180 completed participants (90/arm) are required to provide 80% power to detect a difference of 0.37 in improvement between arms with a two-tailed type 1 error of 0.05. This assumes an overall standard deviation of 0.63 at baseline and 0.81 at 1-month follow-up as found in Bolier et al [16], and a conservative correlation of 0 between baseline and follow-up scores. Considering the higher attrition level found by Bolier et al [16] (37.8%) this study will aim to recruit a sample of 250 participants.

### Randomization

Randomization will occur in blocks of four to ensure approximately equal numbers in each arm. Participants will be asked to read the study information and give their consent online. They will then be directed to create a password-protected account to login to the OWC. After login, participants will be randomized into two groups. The intervention group will have immediate access to The Toolbox, whereas the control group will be allocated to the wait-list for 4 weeks.

### Statistical Analysis

The primary analysis is based on intention to treat, and missing values from all randomized participants will be imputed with 50 samples redrawn from the original data. The primary outcome will be assessed using random effects mixed modeling, with the well-being score as the dependent variable. The independent variables are time, group, and the time × group interaction (product) term, which are clustered over age, gender, geographical location, work status, cultural background, education level, and living circumstances to account for possible correlated readings. Sensitivity analyses and secondary continuous outcomes will be assessed similarly, but also adjusted for potential confounders. All results will be reported with 95% CI and *p* values. A *p* value < 0.05 (two tailed) was taken to be significant. All analyses will be performed using Stata version 13.1 (College Station, TX, USA) [46].

### Cost-Effectiveness Analysis

An economic evaluation will be conducted alongside the trial to estimate the relative cost effectiveness of The Toolbox compared with the wait-list. The evaluation will take the form of a cost-effectiveness analysis based on a primary outcome of cost per unique visit to the OWC. Utility-based outcomes will also be incorporated into the analysis allowing a secondary outcome to be cost per quality-adjusted life year (QALY) gained (based on the AQOL-4D values) [47].

The cost and outcome data will be estimated for every participant within the trial. Costs will be estimated by combining data on resource use associated with both the intervention and control groups with unit costs for each of the resource items. Resource use data will be collected using the resource use questionnaire described earlier. Unit costs (eg, staff wages or opportunity cost of time lost from work, school, or household activities) will be collected from published data sets including those from the Australian Bureau of Statistics and the Australian Government Fair Work Ombudsman. A participant-level analysis will be undertaken to determine the mean costs, increases in number of visits to the OWC, and QALYs for each trial arm. To test the robustness of the economic evaluation results, nonparametric bootstrapping and appropriate sensitivity analyses will be carried out and results will be reported in terms of incremental cost-effectiveness ratios and cost-effectiveness acceptability curves. The final results will be presented in the CONSORT format for reporting randomized trials [48] and the CONSORT-EHEALTH extension [49].

### Results

This trial is funded by the Young and Well Cooperative Research Centre, Country Health South Australia Local Health Network and Flinders University. Recruitment commenced in December 2014 and final results are expected to have been analyzed by the end of 2015. We acknowledge that it may be necessary to implement personalized follow-up strategies (eg, telephone calls to ensure an appropriate number of poststudy measures).

## Discussion

### Principal Findings

The current study differs from previous research in the following key areas: (1) it involves development of a well-being intervention, co-designed with young people using participatory methods; (2) it uses both community and online recruitment approaches to get a representative youth sample of the target population; and (3) it includes an assessment of effectiveness and cost–benefit analysis using a waiting list randomized controlled trial design.

The OWC has been designed as an interactive site to track well-being in a meaningful way for participants. It provides access to self-guided interventions that relate to improvements in well-being and has been designed in collaboration with final users. It is expected that these features will improve engagement rates with the study.

To our knowledge, this is one of the first online interventions designed to improve well-being using self-guided methods specifically targeted at young people. “The Toolbox” has been devised using participatory design methods with ongoing user interaction during all phases of its development. The Toolbox design enables users to choose their own goals. It then specifically recommends suitable apps, while also provides users with a space to rate apps and share their stories with other users. The Toolbox website is personalized, with active components (apps) suggested instead of imposed. It is expected that all of these elements will improve engagement and be reflected in the study outcomes.

The current study will also compare innovative ways to recruit and engage young people using either online advertisements through Facebook and Google, or community-based networks. Such a comparison of recruitment strategies for well-being interventions has not been explored before; most studies recruiting for online well-being interventions have focused exclusively on online recruitment strategies using advertisements [16,18]. The study will subsequently be able to determine whether the cohorts recruited online and offline differ in terms of their general demographic profile, as well as their response to the intervention. The approach is innovative with regard to community-based recruitment, with the intention to develop advertisement/training packages that will be deployed in community organizations using technology (videos, Google Hangouts, etc).

Another novel feature of this study resides in the fact that the intervention is not limited to a clinical population. This opens up opportunities to explore a number of variables that could be highly significant for future interventions; for example “Does well-being improve in the absence or presence of pathology?” and “What are the most effective mechanisms to recruit young people from different backgrounds and engage them with activities to improve their well-being?”

Finally, it is expected that the health economic analysis that will be conducted will determine whether improving well-being in the target population is associated with lower health care costs.

### Limitations

The study has potential limitations in terms of recruitment, adherence, comparison time, and attrition levels. Although research has shown the efficacy of online networking for the recruitment of research participants [23,24], it is known that intrinsic characteristics of young people imply particular challenges regarding adherence in a study promoting well-being. To address these difficulties, recruitment methods have been co-designed with young people and service providers to ensure relevance and adequacy. We also expect to detect differences between recruitment modalities (online vs. community based).

It would have been ideal to administer the resource use questionnaire at baseline and 4 weeks but this will not be possible due to the need to avoid participant burden. The questionnaire will therefore only be applied at 4 weeks with questions focusing on resource use in the intervention and wait-list control groups corresponding to the duration of the trial. Costs will be appropriately extrapolated beyond the 4-week period.

It is not possible to determine how changes in well-being are related to using specific apps. Expected findings of this study will indicate whether accessing purposely built online resources can improve well-being. Given the nature of the study and attempting to improve recruitment and adherence, data will be obtained from just one well-being specific measure. In addition, participant compliance with secondary measures is not mandatory, or assured.

This study attempts to reach the broad population and does not focus on participants with specific profiles, including mental health background history or gender. Although representativeness is pursued mainly through community-based recruitment, it is not guaranteed.

It could be argued that 4 weeks is a relatively short time to assess intervention effects; however, based on input from the current study’s youth reference group it has been found that

1. Young people are more likely to start using apps as soon as they get them and gain full mastery very quickly.
2. Typically, young people will use apps for short periods, and therefore a short intervention could improve retention of the control group.
3. Young people are transient in their interest, which implies that a brief intervention time could yield better adherence.

This study has gained ethical approval by the Social and Behavioural Research Committee of Flinders University under registration number 6478 and is registered in the Australian New Zealand Clinical Trials Registry (ANZCTR); ACTRN: ACTRN12614000710628. It has also gained ethical approval for recruitment by the Department of Education and Child Development of South Australia.
